# Isotopic niche variation in Tasmanian devils *Sarcophilus harrisii* with progression of devil facial tumor disease

**DOI:** 10.1002/ece3.7636

**Published:** 2021-06-06

**Authors:** Olivia Bell, Menna E. Jones, Calum X. Cunningham, Manuel Ruiz‐Aravena, David G. Hamilton, Sebastien Comte, Rodrigo K. Hamede, Stuart Bearhop, Robbie A. McDonald

**Affiliations:** ^1^ Environment and Sustainability Institute University of Exeter Penryn UK; ^2^ School of Natural Sciences University of Tasmania Hobart Tasmania Australia; ^3^ Department of Microbiology and Immunology Montana State University Bozeman MT USA; ^4^ Vertebrate Pest Research Unit NSW Department of Primary Industries Orange NSW Australia; ^5^ Centre for Ecology and Conservation University of Exeter Penryn UK

**Keywords:** devil facial tumor disease, *Sarcophilus harrisii*, sickness behavior, stable isotope analysis, Tasmanian devil

## Abstract

Devil facial tumor disease (DFTD) is a transmissible cancer affecting Tasmanian devils *Sarcophilus harrisii*. The disease has caused severe population declines and is associated with demographic and behavioral changes, including earlier breeding, younger age structures, and reduced dispersal and social interactions. Devils are generally solitary, but social encounters are commonplace when feeding upon large carcasses. DFTD tumors can disfigure the jaw and mouth and so diseased individuals might alter their diets to enable ingestion of alternative foods, to avoid conspecific interactions, or to reduce competition. Using stable isotope analysis (δ^13^C and δ^15^N) of whiskers, we tested whether DFTD progression, measured as tumor volume, affected the isotope ratios and isotopic niches of 94 infected Tasmanian devils from six sites in Tasmania, comprising four eucalypt plantations, an area of smallholdings and a national park. Then, using tissue from 10 devils sampled before and after detection of tumors and 8 devils where no tumors were detected, we examined whether mean and standard deviation of δ^13^C and δ^15^N of the same individuals changed between healthy and diseased states. δ^13^C and δ^15^N values were generally not related to tumor volume in infected devils, though at one site, Freycinet National Park, δ^15^N values increased significantly as tumor volume increased. Infection with DFTD was not associated with significant changes in the mean or standard deviation of δ^13^C and δ^15^N values in individual devils sampled before and after detection of tumors. Our analysis suggests that devils tend to maintain their isotopic niche in the face of DFTD infection and progression, except where ecological conditions facilitate a shift in diets and feeding behaviors, demonstrating that ecological context, alongside disease severity, can modulate the behavioral responses of Tasmanian devils to DFTD.

## Graphical abstract

Tasmanian devils generally maintain their isotopic niche in the face of infection and progression of devil facial tumor disease. The exception was where ecological conditions facilitated a shift in diets and feeding behaviors, such as at Freycinet National Park. Ecological context, alongside disease severity, can modulate the behavioral responses of Tasmanian devils to DFTD.

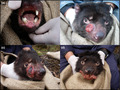

## 1 INTRODUCTION

Animals can alter their behavior in response to infection or disease, whether caused by viral, bacterial, or macroparasitic pathogens, or cancers (Aubert, 1999; Hart, 1988; Vittecoq et al., 2015). Infectious diseases often result in a suite of responses termed “sickness behaviors” that may be associated with variation in host survival, including reductions in movement, food and water intake, aggression, and altered rates of social contacts (Adelman & Martin, 2009; Anderson & Behringer, 2013; Aubert, 1999; Bohn et al., 2016; Hart, 1988; Lopes et al., 2016). Alongside host‐mediated behaviors, some changes in behavior are driven by conspecifics that recognize, and aim to avoid, infected individuals (Behringer et al., 2006; Curtis, 2014), although both sickness behaviors and avoidance can depend on social context (Fairbanks et al., 2015; Lopes et al., 2012). These behavioral changes have implications for population connectivity, predation risk, and transmission of infection through populations and communities (Behringer & Butler, 2010; Bouwman & Hawley, 2010; Lopes et al., 2016).

Compared to the better understood sickness behaviors listed above, relatively little is known about the impact of disease on diet and feeding behaviors (Hite et al., 2020). Sickness behaviors related to diet include reduced food intake, or illness‐induced anorexia, and dietary alterations as a form of self‐medication, or to compensate for the nutritional demands of immune responses (Adamo et al., 2010; Bos et al., 2015; Ghai et al., 2015; Hite et al., 2020; Lee et al., 2006). From an ecological perspective, infection, disease, and associated changes in the social environment create new sets of physiological and ecological constraints for hosts. Food items that were previously considered suboptimal may become preferred. Available resources may also be restricted if the potential for agonistic interactions with healthy conspecifics excludes diseased individuals from preferred food types. Dietary or foraging changes as an ecological response to disease may be particularly likely to occur when a species' diet is closely linked to social or competitive interactions.

**FIGURE 1:**
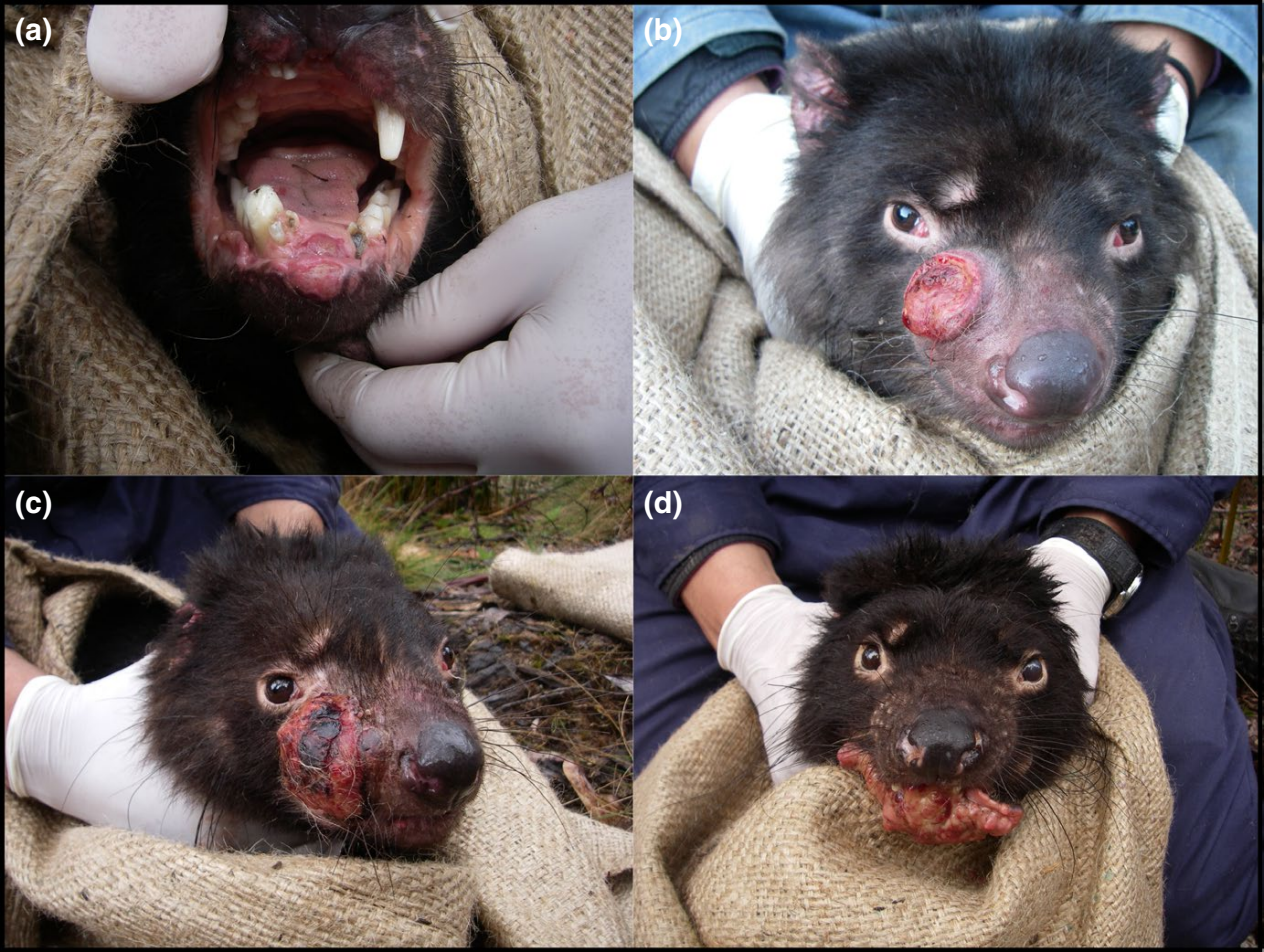
Four individual Tasmanian devils, pictured at four different stages of DFTD progression, with (a) being the earliest disease stage, through to (d) being most severe. DFTD can occur inside or close to the oral cavity (a), which can disrupt the mouth parts and maculatory feeding organs (d). As demonstrated in all images, DFTD tumors often ulcerate, which can lead to secondary infection

Tasmanian devils *Sarcophilus harrisii* are known for intense agonistic interactions, which have indirectly effected severe population declines, following the emergence of a transmissible cancer, devil facial tumor disease (DFTD) (Cunningham et al., 2021; Lazenby et al., 2018; Pearse & Swift, 2006). Transmission of DFTD occurs via direct inoculation of clonal tumor cells from one individual to another during injurious biting behavior, which occurs during agonistic feeding and mating interactions (Hamede et al., 2008, 2013; Hamilton et al., 2019; Pearse & Swift, 2006). DFTD is almost always fatal and the clinical signs present as destructive tumors around the head, neck, and mouth (Figure 1), which lead to death from a combination of metabolic starvation, metastasis, and subsequent organ failure, and secondary infections (Loh et al., 2006). Two evolutionarily distinct transmissible cancers have emerged in Tasmanian devils over a 20‐year period. DFTD was first recognized in 1996 in the northeast of Tasmania, and has spread south and west, resulting in a gradient of disease prevalence and population decline, providing a natural experiment in which to study the impacts of DFTD (Hawkins et al., 2006; Lazenby et al., 2018). A second transmissible cancer, DFT2, emerged in southern Tasmania in 2014, although this is clinically indistinguishable from DFTD and does not yet appear to have spread beyond an area of approximately 550 km^2^ in the d'Entrecasteaux peninsula in southeast Tasmania (James et al., 2019; Pye et al., 2015).

Tasmanian devils have responded to DFTD in their life history and their behavior. Lower conspecific densities in affected populations have resulted in increased growth rates in subadults, probably as a consequence of lower competition for resources, leading to almost 50% of females reaching sexual maturity after their first year, rather than their second year, as in healthy populations (Jones et al., 2008; Lachish et al., 2009). Reduced food competition has also led to reduced dispersal rates in females, probably because of reduced competition (Lachish et al., 2011). Individuals with DFTD display a sustained reduction in conspecific contact rates with disease progression, altering their connectivity in affected populations (Hamilton et al., 2020). Individuals with DFTD also appear to reduce their daily activity; this effect is stronger in males than females, possibly due to the necessity for female devils to travel between den sites and feeding grounds while caring for young (Comte, 2019). Such findings are in line with evidence that diseased females maintain their body condition for longer than males, suggesting they have higher tolerance to DFTD infection, potentially ensuring their survival until any dependent young are weaned (Ruiz‐Aravena et al., 2018).

In response to the progression of DFTD, Tasmanian devils may change their diets to maintain condition and enhance their survival. Devils are facultative scavengers and ordinarily largely eat medium to large herbivores such as Bennett's wallaby *Macropus rufogriseus* and Tasmanian pademelon *Thylogale billardierii*, though scat content analyses show subadult diets to contain lower proportions of these larger species, and higher proportions of birds compared with adults (Andersen et al., 2017; Jones & Barmuta, 1998). Devils are generally solitary, though multiple devils may feed simultaneously at a single carcass (Hamede et al., 2008; Jones, 1995; Pemberton & Renouf, 1993), and feeding is therefore regarded as a source of costly intraspecific interactions and competition in this species. DFTD has high metabolic and physiological costs, evidenced by a reduction in body condition as tumor volume increases (Ruiz‐Aravena et al., 2018). As DFTD tumors grow, they can grossly disrupt the structure of the mouth and jaw, causing necrosis, ulcerations, and secondary infections (Figure 1) (Loh et al., 2006; Pye, Hamede, et al., 2016; Pye, et al., 2016), potentially resulting in a competitive disadvantage for some infected individuals during feeding interactions. Given that devils reduce their social contacts in response to DFTD infection both inside and outside of the mating season (Hamilton et al., 2020), an alteration in diet may facilitate reduced competition and avoidance of conspecific aggression, or be the result of ostracization of diseased individuals by healthy conspecifics. Devils may switch to a diet that can be consumed relatively quickly, easily, and solitarily, carrying a lower risk of costly competition. This could occur via a proportional increase in the consumption of smaller, generally omnivorous, prey, compared with the medium to large, primarily herbivorous, marsupials that usually comprise the bulk of adult devil diets (Andersen et al., 2017; Jones & Barmuta, 2000).

We have applied stable isotope analysis to investigate the effects of DFTD upon Tasmanian devil trophic ecology. Stable isotopes in consumer proteins broadly reflect those of the dietary proteins they have assimilated, subject to alterations as a result of digestion and routing of food sources (Bearhop et al., 2002; DeNiro & Epstein, 1978; Hobson & Clark, 1992). In ecological studies, δ^13^C and δ^15^N are commonly used isotope ratios. Variation in δ^13^C across primary producers provides information on consumer's dietary carbon sources, which may vary among organisms according to movement, foraging grounds, or dietary selectivity (Araújo et al., 2007; Bearhop et al., 2004; Cherel & Hobson, 2007; Crawford et al., 2008; DeNiro & Epstein, 1978). δ^15^N can provide information on trophic position and food web structure, since ^15^N becomes enriched with each trophic level (DeNiro & Epstein, 1981). Trophic enrichment in δ^15^N can also be used to indicate weaning or starvation, as young mammals consuming maternal milk, essentially tissues of their own species, will have high δ^15^N, which reduces through the weaning process (Evacitas et al., 2017; Hobson et al., 1998; Newsome et al., 2009a). Starving animals metabolize their own protein tissues, so are also expected to exhibit elevated δ^15^N values (Hobson et al., 1993). Traditional dietary analysis methods, generally of scat and stomach contents, provide qualitative information on dietary composition, but tend to underrepresent easily digested food items and over‐represent less easily digested items. Stable isotope analysis reflects assimilated diet and can be conducted on inert tissues, such as whiskers and feathers. These tissues may then be subsampled, or sampled on multiple occasions, to build a time series of dietary data representing the period of time the tissue was actively growing. Stable isotope approaches can therefore be particularly useful in building an integrated picture of individual diets and investigating how wild animals respond to change, such as ontogenetic changes in body size and foraging capabilities (Jeglinski et al., 2012; Newsome et al., 2009b), seasonal food availability (Inger et al., 2006), ecosystem fragmentation (Layman et al., 2007), or anthropogenic management activities (Bodey et al., 2010). Our previous stable isotope analysis of Tasmanian devil whiskers showed a significant decrease in δ^13^C and δ^15^N with increasing age, accompanied by a narrowing isotopic niche, of devils as a group and individually, from subadults to adults (Bell et al., 2020). This reduction in trophic level and niche breadth reflects the isotopic effect of weaning, alongside a probable shift in diet from smaller, omnivorous to larger, herbivorous prey species, and a reduction in dietary diversity (Bell et al., 2020). In the context of disease, devil behavioral changes may occur gradually, or might change more swiftly upon individuals reaching an infection load “tipping point” (Szyszka & Kyriazakis, 2013). Stable isotope analysis provides a means of analyzing whether disease progression, not just infection, is associated with changes in foraging ecology.

We used stable isotope analysis of Tasmanian devil whiskers to investigate the impact of devil facial tumor disease on devil foraging ecology in two ways. First, we used a population cross‐sectional study to test whether there is a relationship between tumor volume and whisker isotope values in Tasmanian devils sampled at a range of stages of DFTD progression. We hypothesized that δ^13^C and δ^15^N would change with increasing tumor volume, perhaps via a shift in δ^13^C and an increase in values of δ^15^N associated with a diet comprising fewer large herbivorous marsupials and greater proportions of smaller, more omnivorous species. We considered how this effect could vary based on other ecological variables, including sex, on account of differential tolerance to DFTD (Ruiz‐Aravena et al., 2018), or site. To consider ecological differences among sites that could influence devils' trophic responses to DFTD, we also estimated the relative availability of three of the devils' main prey species: Bennett's wallabies, Tasmanian pademelons, and brushtail possums *Trichosurus vulpecula*, at all sites. Second, we used an individual‐based longitudinal approach to test whether whisker isotope values change in individual devils after DFTD infection, by comparing a set of individuals sampled before and after clinical signs of DFTD were observed, and a comparison set of individuals with no clinical signs of infection, for which we had whisker samples from two time points separated by a comparable interval. We hypothesized that devils that developed DFTD between capture events would exhibit a change in mean and standard deviation δ^13^C and δ^15^N, possibly characterized by an increase in mean δ^15^N associated with feeding at a higher trophic level, and an increase in standard deviation to reflect greater dietary diversity, if diseased devils incorporate previously suboptimal food items.

## 2 METHODS

### 2.1 Field sites

**FIGURE 2:**
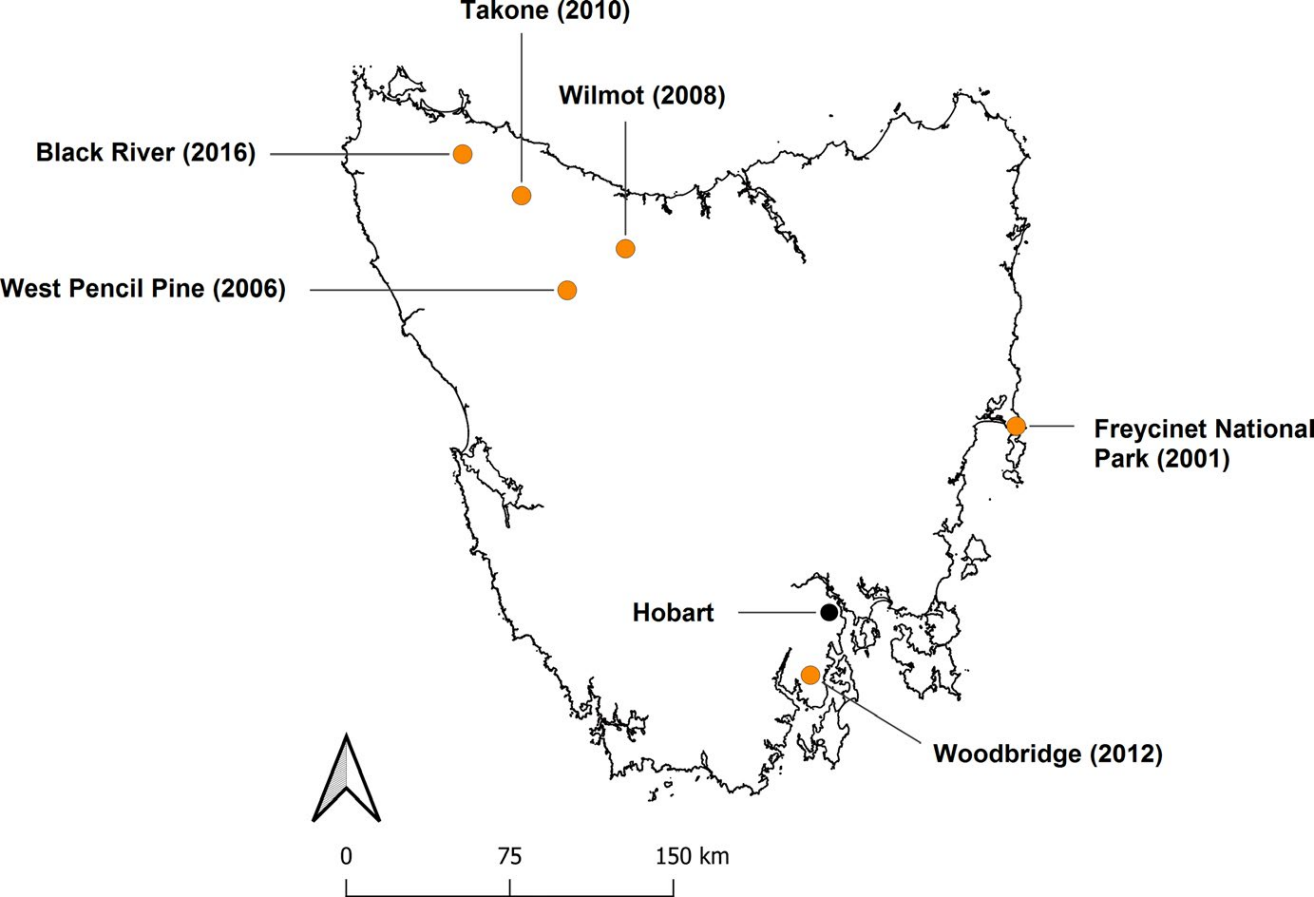
Locations of sites in Tasmania at which Tasmanian devils were sampled. Hobart, the capital city of Tasmania, is shown for reference (black circle). Study sites (orange circles) are labeled with site name, and the year DFTD was first recorded at the site in brackets

To test the effect of DFTD tumor growth, we selected six field sites with varying environments and histories of DFTD infection (Figure 2): Freycinet National Park (−42.107 E, 148.277 S), Woodbridge (−43.131 E, 147.224 S), West Pencil Pine (−41.541 E, 145.823 S), Wilmot (−41.377 E, 146.152 S), Takone (−41.156 E, 145.580 S), and Black River (−40.980 E, 145.263 S). DFTD presence has been recorded at Freycinet since 2001, at West Pencil Pine since 2006, Wilmot since 2008, Takone since 2010, and Black River since 2016. At Woodbridge, DFTD was first recorded in 2012, followed by the emergence of DFT2 in 2014. Freycinet National Park is a coastal site predominantly composed of native dry eucalypt forest. Woodbridge is an area of smallholdings interspersed by native eucalypt woodlands, while Black River, Takone, and Wilmot are commercial eucalypt plantations situated within agricultural landscapes. West Pencil Pine is also a commercial eucalypt plantation, situated close to a large protected area.

### 2.2 Sample collection

Tasmanian devils were caught for sample collection during monitoring surveys carried out at each site at 3‐month intervals. Individuals were identified via subcutaneously implanted microchips (AllFlex© ISO FDX‐B), and age, sex, weight, and head width were recorded. Age was determined using wear on canine and molar teeth, and canine tooth overeruption; this method is accurate because devils sustain predictable high tooth wear and overeruption, and senesce and usually die by five years of age in wild populations (M.E. Jones, unpublished data). Devils were given a standardized birth date of 1 April, as Tasmanian devils give birth 3 weeks after mating in late February/early March (Hesterman et al., 2008).

DFTD status was based on visual diagnosis of clinical signs of the disease (Hawkins et al., 2006). The number and location of tumors present were recorded, and the length, width, and depth of each tumor were measured. Individual tumor volume (mm^3^) was calculated using the formula:

$$\text{ellipsoid volume}=\frac{4}{3}\pi abc$$

where *a*, *b*, and *c* are linear tumor measurements (in this case half the length, width, and depth). Total volume was then estimated as the sum of the individual volumes of all tumors present on an individual devil. This measure has previously been used to successfully describe DFTD severity in Tasmanian devils (Ruiz‐Aravena et al., 2018).

One whisker was collected at each capture for isotope analysis by cutting close to the skin with scissors. In total, whiskers were collected from 112 Tasmanian devils between February 2015 and February 2019 at the six sites (Table 1). Whiskers were stored individually in plastic ziplock bags in a −20°C freezer or fridge prior to laboratory preparation.

**TABLE 1:** Summary of the numbers of individual Tasmanian devils sampled at each site for our population cross‐sectional study and longitudinal study

Site	Sample size	
	Population cross‐sectional study	Longitudinal study
Freycinet National Park	18	7
Woodbridge	8	7
West Pencil Pine	17	NA
Wilmot	24	NA
Takone	10	NA
Black River	17	4
Total	94	18

Note

Our population cross‐sectional study included no individual repeats, while each devil included in our longitudinal study was sampled on two occasions.

### 2.3 Sample preparation

In the laboratory, whiskers were rinsed with distilled water to remove surface contaminants and left to air‐dry, and then placed in a freeze‐dryer for 24 hr. Samples were prepared by chopping small whisker sections into a tin cup until the required analytical sample weight of 0.7 mg ± 0.1 mg was reached.

For the population cross‐sectional study, 94 devils with DFTD were sampled. One sample per individual was taken from the base of each whisker, as this had grown most recently and is therefore most likely to correspond to the tumor volume recorded at the time of capture.

For the individual longitudinal study, we included individuals based on three constraining factors, where individuals must have been free from clinical signs of DFTD when the first whisker was sampled, at least 18 months old at the first sampling occasion to reduce likelihood of age effects masking variation (Bell et al., 2020), and the two whiskers must have been sampled at least 6 months apart, to maximize the likelihood of independence of the two time points. In total, 18 individuals were selected; 10 had DFTD when sampled on the second occasion, while 8 “control” individuals were free of tumors on both sampling occasions. Each whisker was divided into 4 sections (base, 2 middle sections and tip), and each section was subsectioned. One sample per section was taken, by randomly selecting small subsections until the required sample weight was reached, resulting in four samples per whisker.

### 2.4 Stable isotope analysis

All stable isotope analyses were conducted using a Sercon Integra2 elemental analyzer isotope ratio mass spectrometer at the University of Exeter. Stable isotope ratios are expressed as delta (δ) values expressed in parts per thousand, or per mil, (‰) relative to international standards, according to:

$$\delta X=\left[\left(\frac{R_{\text{sample}}}{R_{\text{standard}}}\right)‐1\right]\times 1000$$

where X = ^13^C or ^15^N and *R* = measured ratio of ^13^C to ^12^C, or ^15^N to ^14^N. If the heavy‐to‐light ratio is higher in the sample than the standard, this results in a greater, or “enriched,” δX values. Conversely, heavy‐to‐light ratios that are lower than the standard, lead to lower, or “depleted,” δX values. The standards for δ^13^C and δ^15^N are the Vienna Pee Dee Belemnite and atmospheric nitrogen, respectively; however, other materials calibrated against these standards are routinely used. Our samples were scale‐corrected using the international standards USGS40 and USGS41, with additional internal standards of bovine liver and alanine. Average precision was 0.05‰ ± 0.01 (1 standard deviation ± standard error) for both δ^13^C and δ^15^N.

### 2.5 Statistical analysis

All statistical analyses were conducted in R Version 3.5.2 (R Core Team, 2018).

### 2.6 Population cross‐sectional study

For the cross‐sectional study, we tested the effect of tumor volume on isotope values from basal whisker sections of 94 devils by fitting two linear models using the R package lme4 (Bates et al., 2015), with δ^13^C and δ^15^N as response variables. Our main variable of interest, tumor volume (mm^3^), was included as a fixed effect after we applied a log_10_ transformation to account for positive skew. Age (in months), sex, and a sex​ * tumor volume interaction term were also included. Body condition was added as a fixed effect, as a change in isotope values with changing body condition may be reflective of a change in diet or changing metabolic processes, as animals under nutritional stress exhibit enriched δ^15^N (Hobson et al., 1993). Body condition was estimated using the scaled mass index (SMI) (Peig & Green, 2009):

$$\text{scaled mass index}:{\hat{M}}_{i}=M_{i}{\left[\frac{L_{0}}{L_{i}}\right]}^{b_{\mathrm{SMA}}}$$

where *M_i_* and *L_i_* are the body mass and a linear body measurement of individual *i* (in this case head width), and *L*_0_ is an arbitrary value of *L* (in this case mean head width). To calculate the SMI for each individual, Tasmanian devil mass was first corrected by subtracting tumor mass from the total devil mass (assuming a tumor density of 1.1 g/ml, based on the average density of soft tissues), and then, the SMI was calculated using the corrected devil mass and head width. Site was added to our model as a fixed effect, alongside a site * tumor volume interaction term, as any patterns of isotopic variation may differ based on site‐specific ecological conditions. Year was fitted as a fixed effect, and was an integer, based on yearly trapping cycles from October to October.

Model selection was conducted using the package MuMIn (Bartoń, 2018). To place all variables on the same scale, our predictor variables were rescaled to have a mean of 0 and a standard deviation of 0.5. We then used our global models, built using biologically realistic predictor variables, to create sets of top models where ΔAIC was lower than 2. These top model sets were then averaged, resulting in one final model for δ^13^C and one for δ^15^N. We used the conditional average for model interpretation; as these models avoid shrinkage of model estimates, and are preferable when there is a variable of particular a priori interest (Grueber et al., 2011; Nakagawa & Freckleton, 2011), in this case disease progression.

### 2.7 Species distribution models

Any site‐based differences in the relationship between δ^13^C and δ^15^N values of infected devil whiskers and DFTD tumor volume could be driven by ecological differences among sites, including variation in prey availability. To explore differences in prey availability among the sites, we modeled the relative abundance of three of the main prey species of Tasmanian devils: Bennett's wallabies, Tasmanian pademelon, and brushtail possums (Andersen et al., 2017) across the whole of Tasmania. To do this, we used data from standardized spotlight surveys (see Appendix A), conducted annually from 1985 to 2019, at up to 172 10‐km transects across Tasmania (Figure 5a) (Hocking & Driessen, 1992).

We modeled the spotlight counts for each species using integrated nested Laplace approximation (INLA), fitted using the inlabru R package (Bachl et al., 2019; Illian et al., 2013; Lindgren et al., 2011). For each species, we modeled the count of animals detected per transect in response to explanatory variables for the proportional cover of the four main vegetation classes in Tasmania: wet Eucalypt/rainforest (*%wetEuc*; 28% of Tasmania), dry Eucalypt forests and woodlands (*%dryEuc*; 24%), agricultural land (*%agric*; 23%), and button grass moorlands (*%butGrass;* 9%). We omitted *%dryEuc* from the analysis because it was negatively correlated with *%wetEuc* (Pearson's *r* = −0.65). In addition to simple linear effects, we also modeled nonlinear effects of these covariates (see Appendix A). We accounted for spatial dependency in the spotlight counts, as well as correlations between repeated surveys of transects, with the use of a spatial random field. Following the advice of Illian et al. (2013) for models that include spatial covariates and spatial random fields, we first fitted all combinations of explanatory variables. Then, using the best‐performing covariate model, we tested whether adding a random field improved model fit. Models were compared using the widely applicable information criterion (Watanabe, 2010).

Using the best‐performing models, we produced predictive maps of each species' relative abundance across Tasmania. From the predicted maps, we estimated the relative abundance (±standard deviation) of each prey species, within a buffer around devil trap locations of 3.22 km, which is the radius of the mean 95% kernel density estimate recorded for female devils at Freycinet National Park prior to the first recorded DFTD infection in the area (S. Comte and M. E. Jones, unpublished data). We used female ranges as female devils have larger home ranges than males. For further details of the spatial modeling, see the Appendix A.

### 2.8 Individual longitudinal study

For the longitudinal study of 18 individuals sampled on two occasions, we tested whether DFTD infection results in a shift in individual isotope values, by fitting two linear models, with the response variables as the mean δ^13^C and mean δ^15^N of the whisker sampled on the second capture occasion. We regressed this against the mean δ^13^C and mean δ^15^N of the whisker sampled on the first capture occasion and log_10_‐transformed tumor volume (mm^3^, calculated as above). To test whether DFTD infection results in changes in individual isotopic variation, we then fitted two linear models, with the standard deviation of δ^13^C or δ^15^N of the whisker sampled on the second capture as the response variables. Our models of standard deviation followed the same structure as those of mean δ^13^C and δ^15^N; fixed terms were the standard deviation of δ^13^C or δ^15^N of the whisker sampled on the first capture occasion and log_10_‐transformed tumor volume. Parameter standardization was conducted for all four models using the methods described above. Although standard deviation data tend to be skewed, our data were not strongly skewed and our checks for residual normality, homogeneity of variance, and unduly influential data points did not raise any concerns.

Differences in foraging behavior could lead to differential likelihood of agonistic interactions with conspecifics and subsequent DFTD infection. To test whether isotopic ratios at the first sampling occasion predict subsequent appearance of tumors, we fitted a binomial model with a logit link function, with DFTD infection status at the second sampling occasion as the response variable, and the mean δ^13^C and δ^15^N at the first sampling occasion, and the time in months between sampling events (as likelihood of contracting DFTD may increase with increased time between observations) as predictor variables.

## 3 RESULTS

### 3.1 Population cross‐sectional study

**FIGURE 3:**
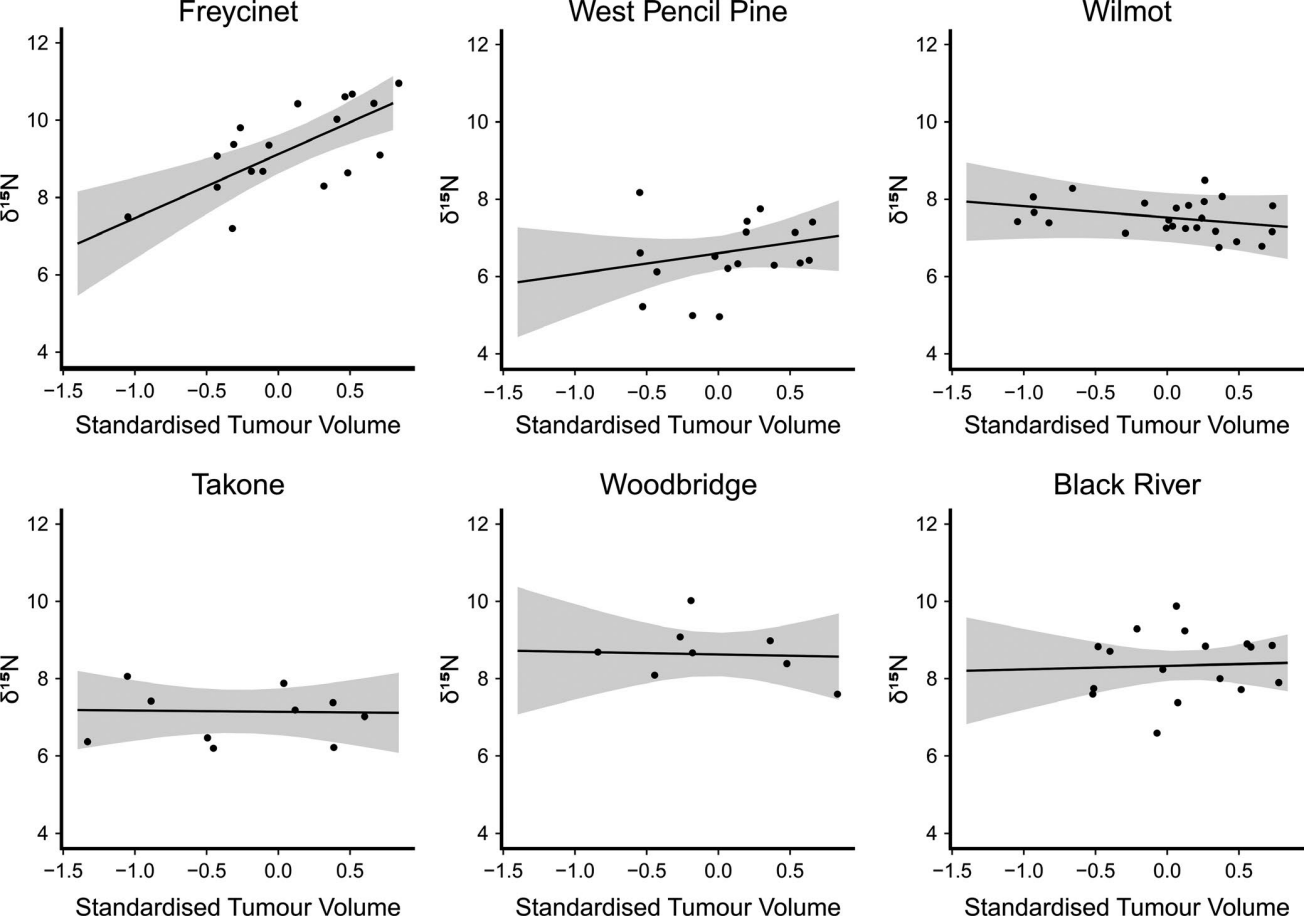
The effect of increasing DFTD tumor volume (log_10_‐transformed and standardized) on δ^15^N values of Tasmanian devil whiskers at six study sites across Tasmania. δ^15^N data are presented with slopes predicted from our standardized linear model. Devils at Freycinet, but not the other sites, show a sharp increase in δ^15^N with increasing tumor volume (estimate = 2.11, 95% CI = 1.67–2.55)

**FIGURE 4:**
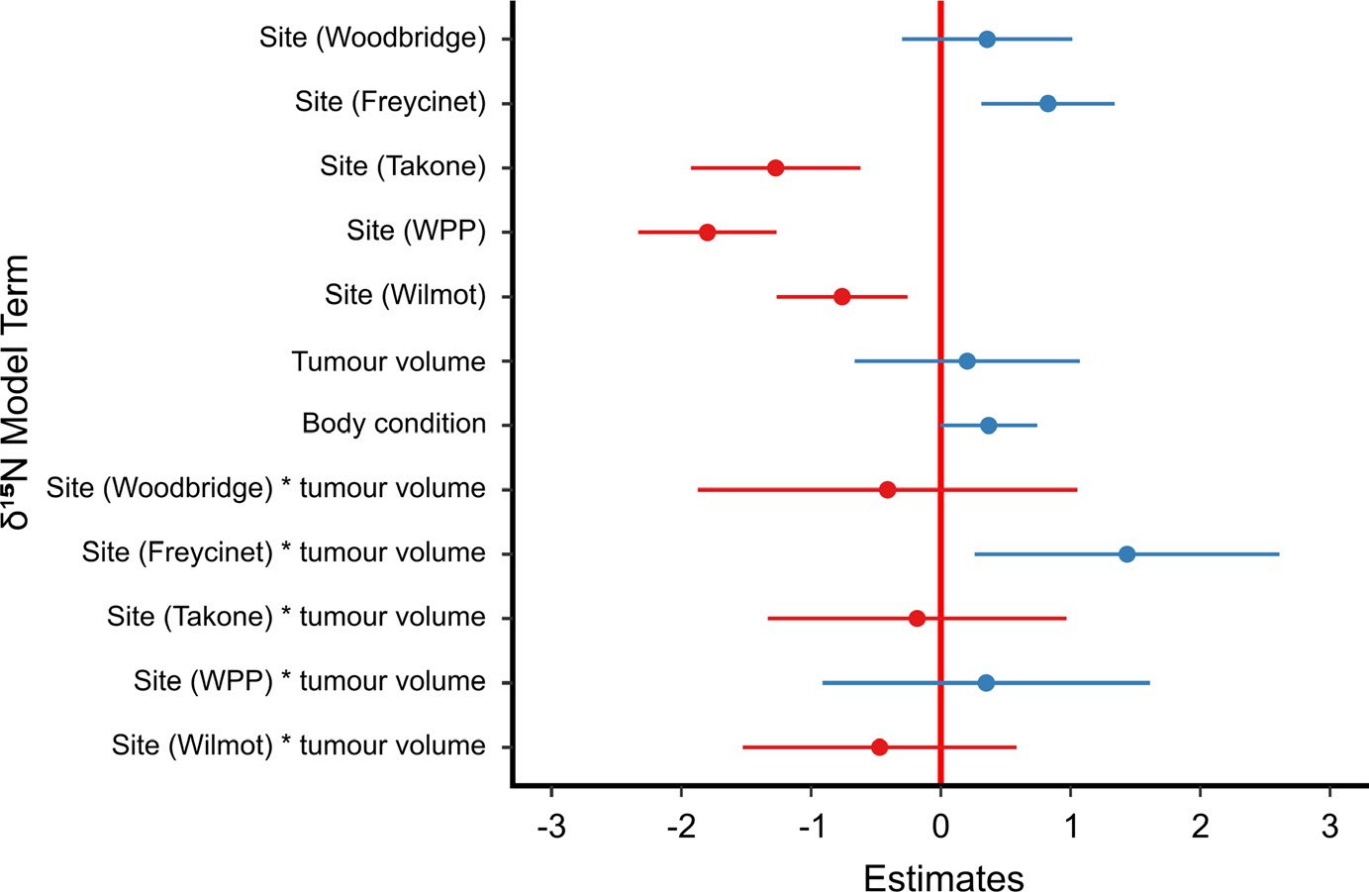
Results from a linear averaged model of variation in stable isotope ratios (δ^15^N) from whisker samples of 94 Tasmanian devils infected with devil facial tumor disease. The site West Pencil Pine is denoted by (WPP). Effect sizes and confidence intervals are presented from our model, produced by averaging two models with a ΔAIC > 2

Tumor volume in our sample of 94 devils with DFTD was strongly skewed toward small volumes. The median volume of tumors was 10,676 mm^3^ (95% quantiles = 204–147,061 mm^3^). Mean δ^13^C of devil whiskers was −23.66‰ (standard deviation = 1.22‰), while mean δ^15^N was 7.89‰ (*SD* = 1.26‰). Two models featured in the top model set for variation in δ^15^N, and four models were in the top set for δ^13^C (Table 2). Prior to model selection, our global models for δ^13^C and δ^15^N had adjusted *R* ^2^ values of 0.71 and 0.63, suggesting a high proportion of the variation in our data is explained by the variables included in our global model. For δ^15^N, there was a strong effect of the site*tumor volume interaction; at Freycinet National Park, but not at the other sites, δ^15^N increased positively with tumor volume (estimate = 1.44, 95% confidence interval = 0.26–2.61) (Figure 3). Tumor volume was not included in our averaged model as a main effect (Figure 4). δ^15^N varied significantly among sites, reflecting variation in the isotopic baseline of different sites. Body condition was also retained in the averaged model, showing a positive trend with increasing tumor volume. For δ^13^C, neither tumor volume nor the site*tumor volume interaction influenced variation among devils, though tumor volume was retained in the average model with a slight negative effect. Site was included in all the top models used to create the averaged model, again reflecting differences in the isotopic baseline of our sites. Age was retained in our averaged model, but with only a slight negative effect of increasing age on δ^13^C. We tested the role of variation in prey availability among sites on isotopic values by replacing site as a stand‐alone variable, and in an interaction term with tumor volume, with our estimates of mean relative abundance for Bennett's wallabies, Tasmanian pademelon, and brushtail possums. However, these models had an AIC estimate at least 2 units higher than models with site; therefore, we retained site as a variable in our model.

**TABLE 2:** Summary of analyses of variation in the stable isotope ratios (δ^13^C and δ^15^N) of whiskers from 94 Tasmanian devils infected by devil facial tumor disease

Response variable	Model variables	Estimate	Standard error	Lower confidence interval	Upper confidence interval	*Z* value	*p* value	Relative variable importance
δ^13^C	Intercept	−23.77	0.15	−24.08	−23.45	147.81	<.001	NA
	Site (Woodbridge)	−0.39	0.29	−0.96	0.18	1.34	.18	1.0
	Site (Freycinet)	2.11	0.22	1.67	2.55	9.45	<.001	
	Site (Takone)	−0.12	0.28	−0.67	0.43	0.43	.67	
	Site (West Pencil Pine)	−0.03	0.23	−0.49	0.43	0.12	.90	
	Site (Wilmot)	−0.95	0.21	−1.36	−0.53	4.49	<.001	
	Tumor volume	−0.19	0.14	−0.46	0.08	1.30	.19	0.45
	Age	−0.21	0.16	−0.52	0.12	1.30	.19	0.43
δ^15^N	Intercept	8.32	0.19	7.94	8.70	43.17	<.001	NA
	Site (Woodbridge)	0.36	0.33	−0.30	1.01	1.07	.29	1.00
	Site (Freycinet)	0.83	0.26	0.31	1.34	3.15	.002	
	Site (Takone)	−1.27	0.33	−1.93	−0.62	3.82	<.001	
	Site (West Pencil Pine)	−1.80	0.27	−2.33	−1.27	6.61	<.001	
	Site (Wilmot)	−0.76	0.25	−1.27	−0.26	3.0	.003	
	Tumor volume	0.20	0.44	−0.66	1.07	0.46	.65	1.00
	Tumor volume: Woodbridge	−0.41	0.74	−1.87	1.05	0.55	.58	1.00
	Tumor volume: Freycinet	1.44	0.59	0.26	2.61	2.40	.02	
	Tumor volume: Takone	−0.18	0.58	−1.33	0.97	0.31	.76	
	Tumor volume: West Pencil Pine	0.35	0.63	−0.91	1.61	0.54	.59	
	Tumor volume: Wilmot	−0.47	0.53	−1.53	0.58	0.88	.38	
	Body condition	0.36	0.18	−0.01	0.74	1.93	.05	0.69

Note

The conditional averaged results for two linear models, with each isotope as a response variable, are presented. Tumor volume was log_10_‐transformed before adding to the model, and all predictors were standardized.

### 3.2 Species distribution models

**FIGURE 5:**
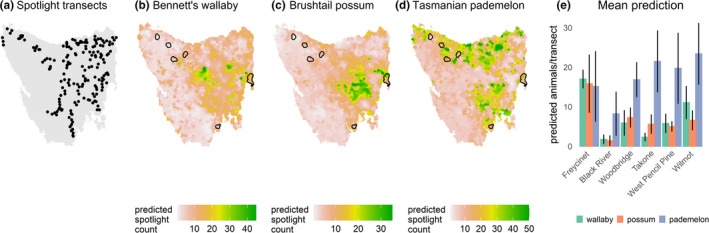
Distribution of (a) spotlight transect surveys, (b–d) species distribution models, and (e) predicted abundances for Bennett's wallabies, brushtail possums, and Tasmanian pademelons. For our study sites (e), predictions are of mean counts of animals per transect (± standard deviation). Species distribution models were created using data from standardized spotlight surveys, conducted annually from 1985 to 2019, at up to 172 10‐km transects across Tasmania

Our top‐performing species distribution models suggest that the prey community differs substantially between Freycinet and our other sites. The model‐estimated relative abundance of the three prey species showed that Freycinet had the highest mean predicted relative abundance of both Bennett's wallabies (17.01 ± 2.36 *SD* predicted animals per transect) and the omnivorous brushtail possums (15.83 ± 7.36 *SD* predicted animals per transect) (Figure 5e). Tasmanian pademelons appear to be relatively consistently abundant across all sites (Figure 5e). The distributions of three major prey species of devils, Bennett's wallabies, Tasmanian pademelons, and brushtail possums, were all influenced by wet eucalypt, agricultural habitat, and button grass moorlands, and the top‐performing model for each species contained a spatial random field (Appendix A, Table A1).

### 3.3 Individual longitudinal study

Of the 18 devils sampled on two occasions to study the longitudinal effects of DFTD on devil isotopic signatures, the 10 individuals that developed DFTD between capture occasions had a median tumor volume of 19,305 mm^3^ (95% quantiles = 602–72,514 mm^3^).

**FIGURE 6:**
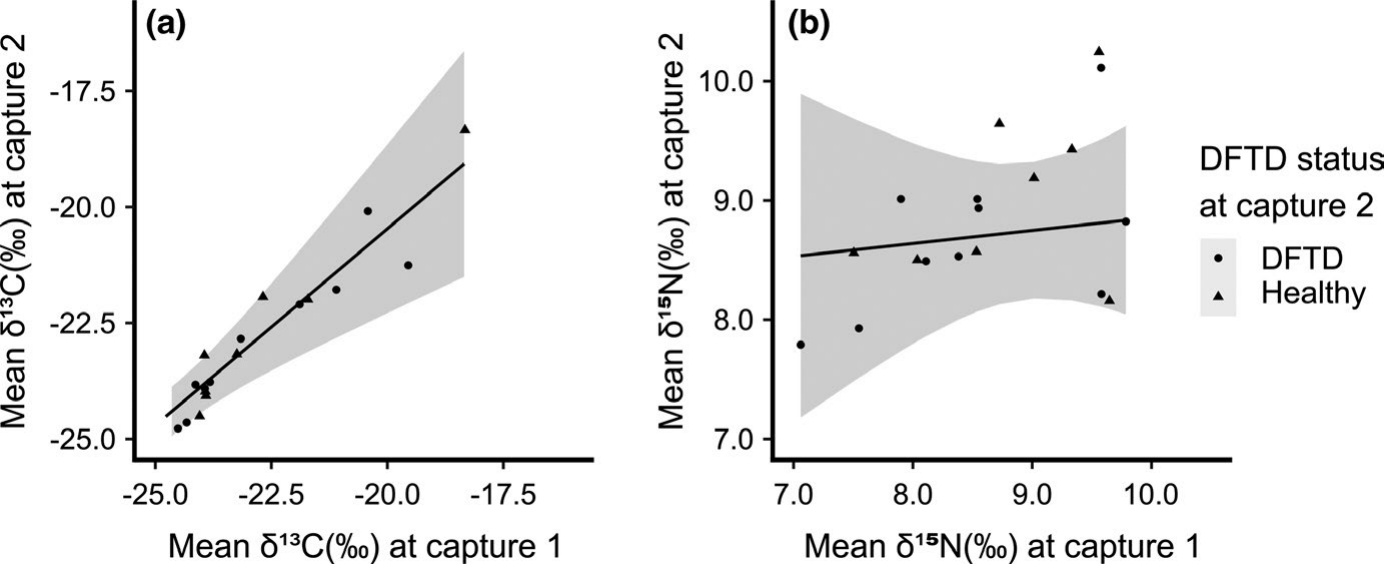
The relationship between (a) mean δ^13^C and (b) δ^15^N of whiskers from 18 Tasmanian devils sampled on two occasions. In both instances, mean isotope values of individuals' whiskers at capture occasion 1 were a significant predictor of mean isotope values at capture occasion 2 (δ^13^C estimate = 3.43, 95% CI = 2.84–4.03, δ^15^N estimate = 0.71, 95% CI = 0.09–1.33). Unstandardized δ^13^C and δ^15^N data are presented with slopes representing model predictions from a linear model

Tumor volume did not explain variation in mean or standard deviation of δ^13^C or δ^15^N at capture occasion 2 (Table 3). Across both δ^13^C and δ^15^N models, the mean ratios at capture occasion 1 were closely correlated with mean ratios at capture occasion 2 (δ^13^C estimate = 3.47, 95% CI = 2.87–4.08; δ^15^N estimate = 0.69, 95% CI = 0.04–1.34; Figure 6). The adjusted *R* ^2^ for our mean δ^13^C model was 0.89, and the adjusted *R* ^2^ for our mean δ^15^N model was 0.19. The standard deviation in δ^13^C at capture occasion 2 was closely correlated with the same metric at capture occasion 1 (estimate = 0.40, 95% CI = 0.05–0.74). Neither standard deviation in δ^15^N at capture occasion 1 nor tumor volume predicted standard deviation in δ^15^N at capture occasion 2. The adjusted *R* ^2^ for our standard deviation δ^13^C and δ^15^N models was 0.23 and 0.17.

**TABLE 3:** Summary of analyses of variation in the mean and standard deviation of stable isotope rations (δ^13^C and δ^15^N) of whiskers from Tasmanian devils sampled before and after detection of clinical signs of devil facial tumor disease

Response variable	Model variables	Estimate	Standard error	Lower confidence interval	Upper confidence interval	*t* value	*p* value
Mean δ^13^C (*t*_2_)	Intercept	−22.70	0.14	−22.99	−22.41	−165.97	<.001
	Mean δ^13^C (*t*_1_)	3.47	0.28	2.87	4.08	12.30	**<.001**
	Tumor volume	0.18	0.28	−0.42	0.78	0.63	.54
Standard deviation of δ^13^C (*t*_2_)	Intercept	0.46	0.08	0.30	0.63	5.98	<.001
	Standard deviation of δ^13^C (*t*_1_)	0.40	0.16	0.05	0.74	2.46	.**02**
	Tumor volume	0.22	0.16	−0.12	0.57	1.39	.18
Mean δ^15^N (*t*_2_)	Intercept	8.84	0.15	8.53	9.15	60.76	<.001
	Mean δ^15^N (*t*_1_)	0.69	0.31	0.04	1.34	2.25	.**04**
	Tumor volume	−0.17	0.31	−0.82	0.48	2.25	.59
Standard deviation of δ^15^N (*t*_2_)	Intercept	0.39	0.03	0.32	0.46	11.66	<.001
	Standard deviation of δ^15^N (*t*_1_)	−0.10	0.07	−0.25	0.04	−1.51	.15
	Tumor volume	0.10	0.07	−0.05	0.25	1.48	.16

Note

Devils were each sampled at two separate time points. 10 individuals developed DFTD between capture occasion 1 (*t*_1_) and 2 (*t*_2_), while 8 remained disease free. The results for four linear models are presented, with the mean and standard deviation of each isotope at the individuals' second capture occasions as response variables. Across both δ^13^C and δ^15^N, tumor volume did not predict mean or standard deviation isotope values at capture occasion 2. Significant *p* values are indicated in bold.

Our binomial model demonstrated that mean δ^13^C and δ^15^N of individuals at the first capture event (while healthy) and the time between capture events did not predict subsequent appearance of DFTD tumors (δ^13^C mean estimate = −0.08, 95% CI = −0.72 to 0.58; δ^15^N mean estimate = −0.45, 95% CI = −1.99 to 0.88; time (in months) estimate = −0.14, 95% CI = −0.40 to 0.03).

## 4 DISCUSSION

Stable isotope ratios in Tasmanian devil whiskers do not, for the most part, vary in response to the progression of DFTD infection. Variation in δ^13^C and in δ^15^N values among infected devils was not associated with tumor volume as a main effect, and neither did mean or standard deviation in δ^13^C or δ^15^N alter after clinical signs of DFTD infection was detected. Tumor volume was, however, related to a pronounced increase in δ^15^N values at Freycinet National Park. The composition of the prey community differed significantly at Freycinet National Park, when compared to all other sites, suggesting that any dietary response by devils to infection may depend on local environmental or ecological conditions.

We predicted that stable isotope ratios of Tasmanian devil whiskers would alter in response to DFTD progression, on the basis that diseased devils might experience reduced energy for foraging, increased difficulty eating, and changes in diet to avoid social contacts (Hamilton et al., 2020; Loh et al., 2006; Pye, Hamede, et al., 2016; Pye, Woods, et al., 2016). Specifically, we suggested that δ^13^C and δ^15^N may increase with increasing tumor volume, to reflect a shift from a diet relying heavily upon herbivorous macropods, such as Bennett's wallabies and Tasmanian pademelons, toward smaller prey, which tend to be omnivorous and are less likely to attract competition. At Freycinet, although we observed no change in δ^13^C, we found δ^15^N values increased significantly with increasing tumor volume, which may reflect an increase in the trophic level of devil food items specifically reflecting a shift from a diet largely comprising herbivorous macropods toward more omnivorous prey species. Our relative prey availability estimates suggest that prey composition at Freycinet differed substantially from our other sites, with higher estimated relative abundance of Bennett's wallabies and brushtail possums, whereas Tasmanian pademelons dominated our other sites. The higher availability of omnivorous prey species at Freycinet, like brushtail possums, could allow devils to prey shift when tumors become larger, which would result in an increase in δ^15^N values. Our results could indicate more broad differences in prey assemblage and availability of other species not modeled here, such as bandicoots, birds, or skink species, which allow devils to transition to a diet richer in omnivores and lower in herbivorous macropods as DFTD infection progresses. Although we do not have data on relative carcass availability between sites, it is reasonable to presume differences in relative abundance of prey species among sites will also lead to differences in the proportion of these carcass types available to scavengers, which may influence the ability of scavengers to shift their diet. Of all our sites, Freycinet is the only site largely comprised of protected areas, and has the most pristine habitat, alongside a drier and warmer climate. This likely leads to differences in relative prey abundance, but other environmental differences, such as predominant foraging habitat, could influence the likelihood that devils will shift their diet with increasing DFTD tumor volume. δ^15^N can increase due to nutritional stress (Hobson et al., 1993); however, if starvation was driving variation in δ^15^N, we would have expected to see a negative, rather than a positive relationship between body condition and δ^15^N (Table 2). We therefore suggest the most likely explanation is that differences in the ecological and environmental context of Freycinet compared with other sites, including prey availability, facilitate changes in devil diets and their isotopic niches as tumor volumes increase.

Given the extent of facial deformation associated with advancing DFTD (Figure 1), it is remarkable that, in sites other than Freycinet, Tasmanian devils retain such consistency in their isotopic signatures with tumor progression. Our longitudinal study showed that mean isotopic signatures, and standard deviation in δ^13^C as a measure of isotopic niche variation, were most strongly predicted by an individuals' prior measures of mean and standard deviation, rather than other ecological predictors, or disease severity, demonstrating the extent to which consistency of inferred diet and dietary variation is maintained among adult devils, regardless of tumor volume. This new information suggests that, while Tasmanian devils exhibit some sickness behaviors in response to DFTD, this generally does not encompass, and is not facilitated by change in the types of food they eat.

Where devils appear not to shift their diets in response to DFTD, they may alter their spatial and social behavior with DFTD progression while maintaining their existing diet and foraging behavior. Outside of the mating season, feeding has been assumed to be a major focus of social and competitive interactions among Tasmanian devils, with direct observational studies of social behavior usually focusing on behavior at carcasses of large prey species (Hamede et al., 2008; Jones, 1995; Pemberton & Renouf, 1993). However, recent video collar evidence found a lower proportion of intraspecific interactions occurred at carcasses than expected, with the majority of interactions occurring while devils were moving (Andersen et al., 2020). The relative importance of social interactions outside of large carcass feeding and mating behaviors may have been underestimated, and as such, foraging may carry a lower relative risk of competitive interactions allowing devils to maintain their usual feeding behavior. Additionally, the preferred food items of devils (including pademelons, wallabies, and possums) may be in high enough abundance that the prospect of intraspecific competition at carcasses is fairly low and there is generally no need for a disease‐related dietary shift. The main prey species of Tasmanian devils are abundant; furthermore, Tasmania has high roadkill density compared with other areas in Australia (Hobday & Minstrell, 2008), providing increased opportunities for facultative scavengers such as Tasmanian devils. The abundance of preferred prey items relative to devil density may vary geographically, resulting in a dietary shift in some areas, but allowing devils to maintain their diet in others.

Devils could maintain the proportional prey composition of their diets, but change aspects of their feeding ecology to accommodate the physical and competitive disadvantages of DFTD infection. For example, infected devils could reduce the overall amount of food they consume. Illness‐induced anorexia can result from a trade‐off between acquiring necessary calories and nutrients, and minimizing energy expended on finding and/or hunting, consuming, and digesting food items (Adamo et al., 2010; McMillan et al., 2018). For Tasmanian devils, reduced food consumption may incidentally result in a reduction in social contact. However, we did not see a sharp increase in δ^15^N in response to DFTD progression or with reduced body condition, which would be expected if food intake reduction and nutritional stress were occurring (Hobson et al., 1993). A further possibility is that devils with DFTD reduce the likelihood of agonistic contacts with other devils by shifting their activity to times of the diel cycle when devil activity is relatively low. Tasmanian devils have been shown to exhibit flexibility in their temporal activity (Cunningham et al., 2019), likely driven by intraspecific competition for carcasses and times of peak herbivore activity. In low‐density sites, peak devil activity is later (around 22:00) than in high‐density sites (peak activity around 18:00) (Cunningham et al., 2019); therefore, infected devils in disease‐affected low‐density sites could avoid competition by foraging in the early evening. Devils could also modify their feeding behavior by abandoning food items more readily on the approach of conspecifics. While direct observations of intraspecific interactions at carcasses have not found strong evidence of dominance hierarchies among devils (Pemberton & Renouf, 1993), larger devils are more likely to displace smaller devils of the same age class (Jones, 1995). This suggests that costly agonistic interactions over carcasses are more likely if an individual is at a competitive disadvantage relative to other devils at a carcass. Diseased devils may therefore have a lower threshold for retreat from carcasses compared with healthier devils, reducing the chance for close agonistic encounters with conspecifics.

We considered whether individuals that contract DFTD, and those that do not, exhibit differences in their foraging that influence their susceptibility to DFTD infection. Tasmanian devils have variable likelihoods of developing DFTD based on their behavior (Hamede et al., 2013); devils with fewer bites are more likely to develop DFTD, predominantly inside the oral cavity, suggesting dominant, aggressive individuals are more at risk (Hamede et al., 2013). We found that isotopic signatures of healthy devils did not predict whether the same individuals subsequently developed DFTD. However, as not all devils were recaptured after the second sampling occasion, we cannot confirm that that our healthy control individuals did not also go on to show symptoms or contract DFTD at a later date or rule out the possibility that individual differences in foraging behavior may influence the likelihood of DFTD infection.

Stable isotope analysis is a robust method of inferring animal diets and ecological niches (Bearhop et al., 2004; Newsome et al., 2007), and its application has enabled us to provide insight into the ecology of individual devils both before and during DFTD progression. However, isotopic signatures and niches are related to, but are not an exact reflection of, diet and ecological niche. Therefore, we cannot exclude the possibility that Tasmanian devils do change their diet with increasing tumor volume, but that stable isotope ratios of whisker samples are not sensitive enough to reveal this change. If different prey species are isotopically similar, a shift in devil diet with increasing tumor volume may not result in a noticeable change in devil isotope signatures. Equally, if prey species are isotopically distinct but vary in their position in isotopic space geographically, stable isotope analysis of devil tissues from multiple sites may not reveal a consistent directional change in isotopic signatures if devils alter their diet with DFTD progression. Furthermore, our ability to capture changes in the standard deviation of δ^13^C and δ^15^N in whiskers before and after DFTD infection depends on whether dietary variation integrates into the whisker at a rate that matches our sampling protocol. If animals consistently eat a broad range of prey items, this could average out within each whisker section, potentially resulting in a low amount of variation between the four whisker sections we analyzed in our longitudinal study. If dietary variation occurs over a longer period of time, variation between whisker sections may be evident.

Sickness behaviors, and individual and population behavioral responses to disease, have implications for immune responses, disease transmission, and disease management (Bouwman & Hawley, 2010; Johnson, 2002; Lopes et al., 2014, 2016; Silk et al., 2019). It is striking that devils with DFTD generally maintain their isotopic niches, given the pathological severity of, and metabolic demands imposed by, cancer. While our data demonstrate that Tasmanian devils largely maintain their isotopic niches as the disease progresses, where ecological conditions permit devils exhibit greater trophic flexibility. This suggests that any sickness behaviors they manifest are dependent as much upon their ecological context as their pathology.

## CONFLICT OF INTEREST

The authors declare no conflict of interest.

## AUTHOR CONTRIBUTIONS

**Olivia Bell:** Conceptualization (lead); data curation (lead); formal analysis (lead); funding acquisition (lead); investigation (lead); methodology (lead); project administration (lead); validation (lead); visualization (lead); writing‐original draft (lead); writing‐review & editing (lead). **Menna E. Jones:** Conceptualization (equal); data curation (equal); investigation (equal); methodology (equal); resources (equal); supervision (equal); validation (equal); writing‐original draft (supporting); writing‐review & editing (equal). **Calum X. Cunningham:** Data curation (supporting); investigation (supporting); methodology (supporting); writing‐review & editing (supporting). **Manuel Ruiz‐Aravena:** Data curation (supporting); investigation (supporting); methodology (supporting); writing‐review & editing (supporting). **David G. Hamilton:** Data curation (supporting); investigation (supporting); methodology (supporting); writing‐review & editing (supporting). **Sebastien Comte:** Data curation (supporting); investigation (supporting); methodology (supporting); writing‐review & editing (supporting). **Rodrigo K. Hamede:** Conceptualization (supporting); data curation (supporting); investigation (supporting); methodology (supporting); resources (supporting); supervision (supporting); writing‐review & editing (supporting). **Stuart Bearhop:** Conceptualization (equal); formal analysis (equal); methodology (equal); software (equal); supervision (equal); validation (equal); writing‐original draft (supporting); writing‐review & editing (equal). **Robbie A. McDonald:** Conceptualization (equal); funding acquisition (equal); investigation (equal); methodology (equal); project administration (equal); resources (equal); supervision (equal); writing‐original draft (supporting); writing‐review & editing (equal).

## ETHICAL APPROVAL

This study was carried out with approval from the University of Tasmania's Animal Ethics Committee (A0015835; A00016789) and the University of Exeter CLES Penryn Ethics Committee (eCORN002473 v2.2).

## Data Availability

Data are available at the Dryad Digital Data Repository at https://doi.org/10.5061/dryad.xwdbrv1d6.

## APPENDIX A

### Methods: Relative abundance of prey species

We modeled the Tasmania‐wide relative abundance of three of the main prey species of devils (Andersen et al., 2017). This included two macropod herbivores, Bennett's wallaby and the Tasmanian pademelon, and the omnivorous brushtail possum. We made use of a dataset of standardized annual spotlight surveys that have been conducted annually from 1985 to 2019 at up to 172 transects across Tasmania (Figure 5), totaling 5,761 surveys (Hocking & Driessen, 1992). Each transect follows a 10‐km segment of road, with one person operating a handheld spotlight and another recording sightings of all wild animals (Hocking & Driessen, 1992). We considered the count of each species per transect as a measure of relative abundance.

We modeled the distributions of these prey species using integrated nested Laplace approximation (INLA). INLA is a computationally efficient method for Bayesian inference with spatial data. INLA facilitates the use of Gaussian random fields to model spatial dependence between observations (Bachl et al., 2019). Using INLA, a Gaussian random field is approximated using the solution to a stochastic partial differential equation (SPDE) (Lindgren et al., 2011), which requires discretizing space into a tiling of adjacent triangles, known as a mesh (Bachl et al., 2019). We constructed the mesh to have maximum interior edge lengths of 10 km (matching the scale of the spotlight transects). To avoid problematic boundary effects, we extended the mesh beyond the boundaries of Tasmania, using larger edge lengths (30 km) to reduce fitting time (Bakka et al., 2018). We used a Matérn correlation structure for the SPDE (priors = 0.1 probability that the range is less than 10 km and a 0.5 probability that the standard deviation was greater than 10 km). See Lindgren et al. (2011) for a fuller explanation of SPDEs and INLA. We fitted the models using the “inlabru” R package (Bachl et al., 2019), which builds upon the R‐INLA package (Illian et al., 2013).

We constructed four spatial covariates reflecting the percentage cover of the four main vegetation classed in Tasmania: wet Eucalypt/rainforest (*%wetEuc*; 28% of Tasmania), dry Eucalypt forests and woodlands (*%dryEuc*; 24%), agricultural land (*%agric*; 23%), and button grass moorlands (*%butGrass;* 9%). Using a raster (cell size = 1 km^2^) of the TasVeg 3.0 GIS layer (Department of Primary Industries Parks Water & Environment, 2014), we extracted the mean proportional cover of each vegetation class within a 5‐km^2^ area around each spotlight transect (for further details, see Cunningham et al., 2021). We omitted *%dryEuc* from the analysis because it was negatively correlated with *%wetEuc* (Pearson's *r* = −0.65). In addition to simple linear effects, we modeled nonlinear effects of these covariates using a one‐dimensional Matérn SPDE with five evenly spaced B‐spline knots.

We followed the advice of Illian et al. (2013) for selecting models that include spatial covariates and spatial random fields, which can compete for explanatory power. We first aimed to select the most informative environmental covariates. For each species, we tested whether linear or nonlinear effects of vegetation covariate produced the best model fit. Next, we tested all simpler combinations of those environmental covariates, comparing models using the widely applicable information criterion (Watanabe, 2010). Then, we added a spatial random field to the best covariate model. This has two purposes: first, to model spatial dependency not captured by the vegetation covariates and, second, to account for correlations between repeated surveys at each transect. Finally, we compared all models using WAIC and, from the best model, produced predictive maps of relative abundance across Tasmania (“predict” function of “inlabru,” which takes many draws from the model posteriors).

**TABLE A1:** Model selection table for the species distribution models of prey species

Order model fitted	WAIC	Delta.WAIC	Model
Bennett's wallaby			
12	30,569.7	0.0	f(wetEuc) + f(agric) + butGrass +random field
3	32,756.4	2,186.7	f(wetEuc) + f(agric) + butGrass
4	32,759.0	2,189.3	f(wetEuc) + f(agric) + f(butGrass)
2	32,861.9	2,292.2	wetEuc +f(agric) + butGrass
1	32,879.1	2,309.3	wetEuc +agric + butGrass
6	32,883.4	2,313.7	f(wetEuc) + f(agric)
5	33,011.8	2,442.1	f(wetEuc) + butGrass
8	33,107.1	2,537.4	f(wetEuc)
7	33,941.7	3,372.0	f(agric) + butGrass
10	34,008.2	3,438.5	butGrass
9	34,135.5	3,565.8	f(agric)
11	34,199.3	3,629.6	null
Tasmanian pademelon			
12	39,345.9	0.0	f(wetEuc) + f(agric) + f(butGrass) + random field
4	41,898.3	2,552.4	f(wetEuc) + f(agric) + f(butGrass)
3	41,904.8	2,558.9	f(wetEuc) + f(agric) + butGrass
2	41,908.8	2,562.9	wetEuc +f(agric) + butGrass
6	41,914.6	2,568.7	f(wetEuc) + f(agric)
7	41,922.3	2,576.4	f(agric) + f(butGrass)
9	41,938.1	2,592.2	f(agric)
1	42,110.0	2,764.1	wetEuc +agric + butGrass
5	42,573.0	3,227.1	f(wetEuc) + f(butGrass)
10	42,651.9	3,306.0	f(butGrass)
8	42,657.7	3,311.8	f(wetEuc)
11	42,746.9	3,401.0	null
Brushtail possum			
12	30,388.6	0.0	f(wetEuc) + f(agric) + f(butGrass) + random field
4	33,289.6	2,901.1	f(wetEuc) + f(agric) + f(butGrass)
6	33,404.1	3,015.5	f(wetEuc) + f(agric)
5	33,404.9	3,016.3	f(wetEuc) + f(butGrass)
3	33,406.0	3,017.4	f(wetEuc) + agric +f(butGrass)
2	33,417.8	3,029.2	f(wetEuc) + agric +butGrass
8	33,568.3	3,179.7	f(wetEuc)
1	33,691.8	3,303.2	wetEuc +agric + butGrass
7	33,960.4	3,571.8	f(agric) + f(butGrass)
9	34,177.3	3,788.7	f(agric)
10	34,214.8	3,826.2	f(butGrass)
11	34,557.5	4,168.9	null

Note

“Order model fitted” shows the order in which the models were fitted, which followed a model selection approach that first aimed to select the important environmental covariates, and then test whether adding a spatial random field improved model fit. Models were ranked according to the widely applicable information criterion (WAIC), with Delta.WAIC showing the difference between the best model. We tested linear (e.g., “wetEuc”) and nonlinear (e.g., “f(wetEuc)”) effects of each vegetation covariate.
